# The Determination of Polymyxin B in Critically Ill Patients by the HPLC-MS/MS Method

**DOI:** 10.1155/2023/6674009

**Published:** 2023-04-07

**Authors:** Yirong Wang, Jingchun Chen, Jinpan Du, Liming Lei, Boxin Zhao, Yunpeng Bai, Dong Chen, Xipei Wang, Chunbo Chen

**Affiliations:** ^1^School of Biology and Biological Engineering, South China University of Technology, Guangzhou 510006, Guangdong, China; ^2^Department of Intensive Care Unit of Cardiovascular Surgery, Guangdong Cardiovascular Institute, Guangdong Provincial People's Hospital, Guangdong Academy of Medical Sciences, Guangzhou, China; ^3^Department of Pharmacy, Nanfang Hospital, Southern Medical University, Guangzhou, China; ^4^Center of Scientific Research, Maoming People's Hospital, Maoming 525000, China; ^5^School of Medicine, South China University of Technology, Guangzhou 510006, China; ^6^Research Center of Medical Sciences, Guangdong Provincial People's Hospital, Guangdong Academy of Medical Sciences, Guangzhou, Guangdong, China; ^7^Guangdong Provincial Key Laboratory of Clinical Pharmacology, Guangdong Cardiovascular Institute, Guangdong Provincial People's Hospital, Guangdong Academy of Medical Sciences, Guangzhou, Guangdong, China; ^8^Department of Critical Care Medicine, Shenzhen People's Hospital, The Second Clinical Medical College of Jinan University, The First Affiliated Hospital of South University of Science and Technology, Shenzhen 518020, China; ^9^Department of Emergency, Maoming People's Hospital, Maoming 525000, China

## Abstract

Polymyxin B (PB) is a dose-dependent drug used to treat multidrug-resistantgram-negative bacteria, for which a suitable method is needed to determine clinical samples. A simple, economical, and efficient high-performance liquid chromatography-mass spectrometry (HPLC-MS/MS) method was developed and validated for polymyxin B1 (PB1), polymyxin B1-Ile (PB1-I), polymyxin B2 (PB2), and polymyxin B3 (PB3) in human plasma. Chromatographic column was Waters BEH C18 column (2.1 × 50 mm, 1.7 μm). Phase A was water with 0.2% formic acid (FA), and phase B was acetonitrile containing 0.2% FA. The elution method is gradient elutio. The total analysis time was 5 min. The pretreatment method involved protein precipitation using acetonitrile containing 0.2% trifluoroacetic acid and 0.1% FA as the precipitant. The recovery rate was 92–99%. The total quantity of PB1 and PB1-I was measured in the linear range of 100–8000 ng/mL. Simultaneously, the total amounts of PB2 and PB3 were measured in the linear range of 11.9–948.5 ng/mL. This validated method was successfully applied to the pharmacokinetics of PB in critically ill patients.

## 1. Introduction

Polymyxin B (PB) is a lipopeptide antibiotic extracted from the fermentation products of *Bacillus polymyxa* [1]. In clinical practice, considered the “last line of defense,” it is primarily used for infections caused by gram-negative bacteria with multidrug resistance, such as *Pseudomonas aeruginosa*, *Acinetobacter baumannii*, and *Klebsiella pneumoniae* [2, 3]. PB is a dose-dependent drug discovered in the 1950s and has significant adverse effects on the kidney, which has impact on critically ill patients [3–6]. Therefore, it is important to maintain the drug concentration within the therapeutic window. Therefore, therapeutic drug monitoring (TDM) of PB is necessary for clinical practice.

The major components of PB are polymyxinB1 (PB1), B1-Ile (PB1-I), B2 (PB2), and B3 (PB3) [7–9]. Most studies have measured the concentrations of PB1 and PB2 [10–12]. However, in several pharmacopeias, the total content of PB1, PB1-I, PB2, and PB3 in the dried product should not be less than 80% [13–15]. Therefore, it was necessary to measure the sum of the four components. In previous studies, thin-layer chromatography, high-performance liquid chromatography (HPLC), and high-performance liquid chromatography-mass spectrometry (HPLC-MS/MS) have been used for the determination of PB1 and PB2 in plasma [11, 16–19]. HPLC-MS/MS has been the most commonly used method in the past decade owing to its convenience and accuracy in quantitative analysis [20]. Several studies have used HPLC-MS/MS to detect the PB content in human plasma, but only one has measured four components [10, 12, 19, 21, 22]. Hee KH [19] described a method for the determination of PB1, PB1-I, PB2, and PB3, but the run time was long (7.5 min) and the recovery rate using the protein precipitation method (PPE) was low (53–76%).

Pretreatment is necessary to quantify plasma samples using HPLC-MS/MS. Among the pretreatment methods, PB, PPE, and solid-phase extraction (SPE) are commonly used. Several studies measured PB1, PB1-I, and PB2 using SPE, and the recovery rate was ∼60% [12, 21, 22]. However, SPE is expensive and complex. Covelli et al. [10] extracted PB1 and PB2 from human plasma with acetonitrile (ACN) containing 2.0% trifluoroacetic acid (TFA) and yielded higher recovery (93.5–101.2%) with a run time as long as 20 min. However, the pretreatment process was complex, including the processes of extraction, nitrogen blowing, and re-dissolution, and the run time was long. A simple, economical, and efficient HPLC-MS/MS method is required to measure the concentrations of PB1, PB1-I, PB2, and PB3.

In this study, PPE was used to pretreat human plasma. ACN with 0.2% TFA and 0.1% formic acid (FA) as the extraction solution, the content of PB1, PB1-I, PB2, and PB3 was determined, which was more suitable for TDM.

## 2. Materials and Methods

### 2.1. Chemicals and Reagents

Polymyxin B sulfate (purity: 88.5%, lot 1116937) and polymyxin E (PE) sulfate (purity: 93.7%, lot 137369) were purchased from *Dr.Ehrenstorfer GmbH* (Augsburg, Germany). For the standard PB sulfate, the sulfate content was 14.9%. The mixture of PB1, PB1-I, PB2, and PB3 was 81.7, 7.7, 9.3, and 1.3%, respectively. LC-MS-grade ACN was purchased from *Thermo Fisher Scientific* (Massachusetts, USA). Formic acid was obtained from *Sigma-Aldrich* (St. Louis, MO, USA). HPLC-grade trifluoroacetic acid was obtained from *MACKLIN* (Shanghai, China).

### 2.2. Equipment

The LC-MS/MS system consisted of an Agilent 1260 HPLC system equipped with a cooled autosampler (4°C) and an Agilent 6460 electrospray ionization-triple quadrupole mass spectrometer (Agilent Technologies, USA). The chromatographic column was BEH C18 (Waters, 2.1 × 50 mm, 1.7 *μ*m) (Waters Corporation, USA). Ultrapure water was prepared using the Milli-Q Direct 8 (E. Merck, Darmstadt, Germany) water purification system. A Heraeus Multifuge X1R (Thermo Fisher Scientific, USA) high-speed refrigerated centrifuge was used for the centrifugation. SHIMADZU-AUW120D (Shimadzu Corporation, Japan) was used for the weighting.

### 2.3. HPLC-MS/MS Conditions

#### 2.3.1. HPLC Conditions

A Waters BEH C18 column (2.1 × 50 mm, 1.7 *μ*m) was used for the separation of PB. The column temperature was maintained at 35°C. Mobile phase A was water with 0.2% FA (v/v). Mobile phase B was ACN containing 0.2% FA (v/v). Gradient elution was adopted in the experiment: 0.0–0.5 min, 5% B; 0.5–1.0 min, 5–60% B; 1.0–1.5 min, 60% B; 1.5–2.0 min, 60–90% B; 2.0–2.5 min, 90% B; and 2.5–3.2 min, 90–5% B. The posttime was 1.8 min to reach equilibrium. The flow rate was 0.4 mL/min. The injection volume was 5 *μ*L.

#### 2.3.2. Mass Spectrometric Conditions

The MS was based on the multiple reaction monitoring mode (MRM) and positive ionization mode. The precursor ion was [M+2H]^2+^ for PB and PE2 (internal standard, IS). Quantification ion pairs were PB1/PB1-I: 602.7/101.1; PB2/PB3: 595.7/101.1; and PE2: 578.7/101.1. Dwell was 50 for all components. For PB1/PB1-I, the fragmentor was 196, and collision energy (CE) was 37 volts (V); for PB2/PB3, they were 181 and 37 V; for PE2, they were 130 and 35 V.

### 2.4. Preparation of Stock and Working Solution

Standard and IS stock solutions were prepared in Milli-Q water containing 1% FA (v/v) at 1 mg/mL (with all substances) and subpacked in EP tubes. Working solutions were diluted from the stock solution. The concentrations of PB1 in the PB1-I's working solution were 160, 120, 100, 48, 24, 12, 6, and 2 *μ*g/mL. The quality control (QC) working solutions were 140, 80, 20, and 4 *μ*g/mL. The IS solution, polymyxin E2, was diluted in 1% FA water to 7.5 *μ*g/mL. All solutions were stored at −80°C.

### 2.5. Preparation of Calibration Samples and QC Samples

Blank plasma (190 *μ*L) and 10 *μ*L working solution were mixed to prepare calibration curves of 8000, 6000, 5000, 2400, 1200, 600, 300, and 100 ng/ml for PB1/PB1-I, and 948.5, 711.4, 592.8, 284.6, 142.3, 71.1, 35.6, and 11.9 ng/mL for PB2/PB3. Similar to the QC standards, for PB1/PB1-I, the low QC (QCL), medium QC 1 (QCM1), medium QC 2 (QCM2), and high QC (QCH) were 200, 1000, 4000, and 7000 ng/ml, respectively. For PB2/PB3, QC standards were 23.7, 118.6, 474.3, and 830.0 ng/ml. First, a 100 *μ*L calibration sample or QC sample was removed; 10 *μ*L IS solution, 200 *μ*L ACN with 0.1% FA (v/v), and 0.2% TFA (v/v/v) were added successively. The samples were vortexed for 2 min to make precipate the protein fully, then centrifugated at 18800 g at 4 ℃ for 15 min. About 200 *μ*L of the supernatant was sucked and transferred into 96-well plates for analysis.

### 2.6. Extraction Using PPE

Volume of 200 *μ*L ACN with 0.2% TFA and 0.1% FA was added to a 100 *μ*L plasma sample containing 10 *μ*L IS solution, vortexed for 2 min, and centrifugated at 18800 g at 4°C for 15 min. The supernatant was then transferred to a 96-well plate for analysis.

### 2.7. Method Validation

Method validation was based on the Chinese Pharmacopoeia (2020) and the International Council for Harmonisation of Technical Requirements for Pharmaceuticals for Human Use (ICH).

#### 2.7.1. Selectivity and Matrix Effect

Six blank matrices obtained from different individual sources, one high-fat matrix obtained from volunteers with abnormally high triglyceride levels, and the hemolytic matrix prepared by adding hemolytic whole blood (2%, v/v) to the blank matrix were selected to comprehensively evaluate the selectivity and matrix effect of this method. For selectivity, the lower limit of quantification (LLOQ) and blank-level samples were used for evaluation. Eight blank samples were prepared in the above matrices by adding 15 *μ*L solvent and 200 *μ*L precipitant to 95 *μ*L of different matrices. LLOQ samples were prepared with working solutions of LLOQ, IS solution, and matrices. The responses attributable to interfering components in the retention time should not be more than 20% of PB and not more than 5% of IS in the LLOQ sample of each matrix. The matrix effect was evaluated by analyzing at least three replicates at low and high QC concentrations. The QCL and QCH of plasma samples were prepared using their working solutions with the eight matrices in QC sample preparation. QCL and QCH of the solvent samples were prepared using 5 *μ*L working solution, 95 *μ*L solvent, 10 *μ*L IS solution, and 200 *μ*L precipitant. The ratio of analyte and IS in the matrices and solvent, respectively, is calculated as the matrix factor (MF) of the analyte and IS. The IS‐normalized MF was then calculated to evaluate the matrix effect. The coefficient of variation (CV) of the IS‐normalized MF of the eight matrices should not be greater than 15%.

(1) $$\begin{array}{c} MF\;of\;analyte=\frac{\left(peak\;area\;of\;analyte\;in\;the\;matrix\right)}{\left(peak\;area\;of\;analyte\;in\;solvent\right)}\text{,}\\ MF\;of\;IS=\frac{\left(peak\;area\;of\;IS\;in\;the\;matrix\right)}{\left(peak\;area\;of\;IS\;in\;solvent\right)}\text{,}\\ IS‐normalized\;MF=\frac{\left(MF\;of\;the\;analyte\right)}{\left(MF\;of\;IS\right)}\text{.} \end{array}$$

#### 2.7.2. Extraction Recovery

The extraction recovery was calculated by the peak area ratios of samples recovered from plasma, extracted blank plasma, and IS spiked with the same concentrations of PB. Samples at four concentrations were analyzed in triplicates.

(2) $$\begin{array}{c} extraction\;recovery=\frac{\left(peak\;area\;of\;the\;analyte\;added\;before\;extraction/peak\;area\;of\;IS\right)}{\left(peak\;area\;of\;the\;analyte\;added\;to\;the\;extracted\;supernatant/peak\;area\;of\;IS\right)}*100\%\text{.} \end{array}$$

#### 2.7.3. Calibration Curve and Carry-Over

Eight calibration-level samples, a blank sample, and a zero sample were used to establish the curve. The preparation was the same as that described in Section 2.6. The upper limit of quantification (ULOQ) and LLOQ are shown in the curve. The LLOQ is the lowest point of the curve, whereas the ULOQ is the highest. Fitting the curve by the least square regression analysis, the accuracy of the LLOQ should be within ±20%; other calibration samples should be within ±15%. Carry-over was assessed using blank samples after calibration at the ULOQ. Compared with the LLOQ, the area of the analyte should not be greater than 20 or 5% for the IS.

#### 2.7.4. Accuracy and Precision

Accuracy and precision were determined using five QCs: LLOQ, QCL, QCM1, QCM2, and QCH. Five replicates at each concentration level were paralleled for each run. The between-run accuracy and precision were evaluated in three runs over two days. The accuracy of the LLOQ should be within ±20% and that of other QCs should be within ±15% overall. The precision of the LLOQ should not exceed 20%; the other QCs should not exceed 15% overall.

#### 2.7.5. Dilution Integrity

During the investigation of dilution integrity, the dilution QC concentration was 10000 ng/mL, which was diluted with blank plasma. The two dilution factors investigated were two and four, respectively. There were five replicates for each dilution factor. The mean accuracy of the dilution QC samples should be within ±15%; the precision should not exceed 15%.

#### 2.7.6. Stability

QC samples (QCL, QCM1, QCM2, and QCH) were used to investigate the stability of the stock solutions, working solutions, and samples. Each QC was set with three parallels. The stability of plasma samples stored at room temperature (25°C) for 4 h, at 4°C in a refrigerator for 24 h, at −20°C for 37 days, and at −80°C for 97 days was investigated. The freeze-thaw stability of −20°C and −80°C freeze-thaw cycles at three times each was investigated. The stability of the stock and working solutions stored at −80°C for 97 days was investigated. The accuracy of the quality control sample should be within ±15% of the nominal concentration; the precision should be within 15%.

### 2.8. Application to the TDM and Population Pharmacokinetics (PPK) Study

Plasma samples were collected from critically ill patients who had received at least a third dose of polymyxin B in EDTA-K2 blood collection tubes. Blood samples were collected at seven time points: 10 min before drug administration, 5 min, 1, 2, 4, and 8 h after infusion, and 10 min before the next drug administration. Blood samples were centrifuged at 996 g at 4°C for 10 min, separated into EP tubes, and frozen at −80°C. This study was approved by the institutional review board of Guangdong Provincial People's Hospital. A total of 350 clinical plasma samples were collected. The PB content was measured using the method developed in this study. The results were used to study the population pharmacokinetics (PPK). Alternatively, this method could be used for clinical drug concentration monitoring. Plasma samples of the peak and valley concentrations of PB were collected and measured using HPLC-MS/MS after validation.

## 3. Results and Discussion

### 3.1. LC-MS/MS Method Development

**Figure 1:**
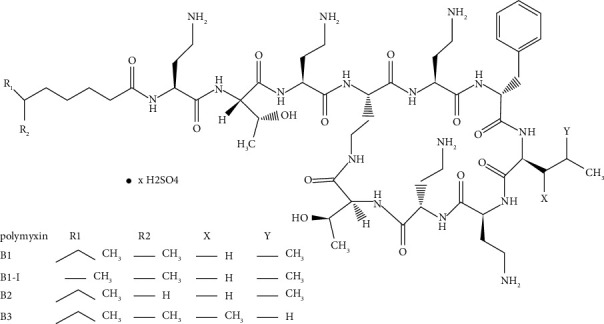
The structure of PB.

**Figure 2:**
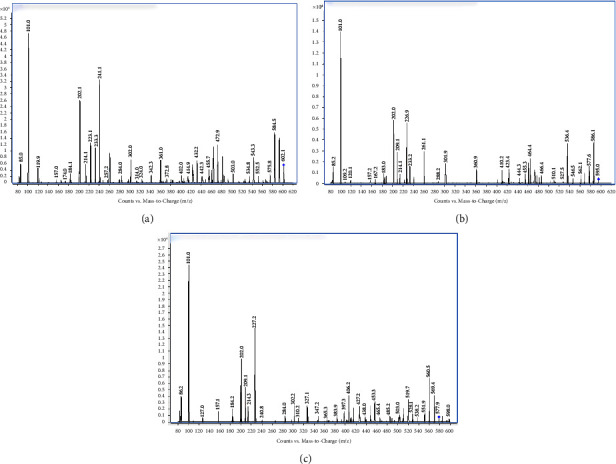
The product iron scan of PB1/PB1-I (a), PB2/PB3 (b), and PE2 (c) showing fragmentation patterns of PB1/PB1-I, PB2/PB3, and PE2 (IS).

**Figure 3:**
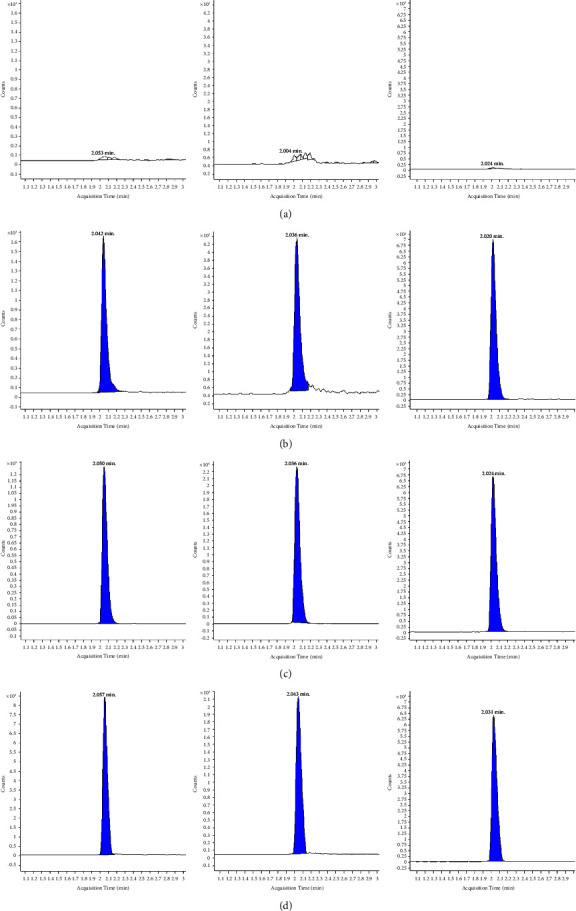
The chromatograms of PB and IS in human plasma. The left is PB1/PB1-I; the middle is PB2/PB3; and the right is IS. (a) Blank plasma sample; (b) plasma standard of LLOQ; (c) plasma standard of ULOQ; (d) clinical plasma sample.

PB standards include PB1, PB1-I, PB2, and PB3. In this method, PB1 and PB1-I were not separated by chromatography, similar to PB2 and PB3. PB's structural formula is shown in Figure 1. PB1 and PB1-I are isomers, and the quantitative ion pair was the same at 602.7/101.1. Based on previous reports [19, 20], it was considered that they could be determinated as one peak. Similarly, PB2 and PB3 were isomers. The quantitative ion pair was 595.7/101.1, suggesting that they could be determinated as one peak too. The PB content is the sum of the four components. The precursor ion of PB1/PB1-I was [M+2H] ^2+^ 602.7. The iron product is shown in Figure 2. Iron 101.0 was selected, which was common to PB1 and PB1-I, and had the highest response. The precursor ion of PB2/PB3 was 595.7, a form of [M+2H] ^2+^. The product scan of iron is shown in Figure 2; 101.0 was selected for the same reasons. The product scan of IS is shown in Figure 2; the iron pair was 578.7/101.1. The typical peak shapes of the blank, plasma standard of LLOQ and ULOQ, IS, and clinical samples are shown in Figure 3. Overall, it is feasible to measure the PB components of the same mass together as the peak shape of each concentration is good, meeting the quantitative requirements.

For the measurement of clinical samples, PPE is simple, more convenient, and more economical than SPE. Therefore, PPE was adopted in this method. Comparing the extraction recovery and the peak area of ACN, ACNe containing 0.1% FA, ACN with 0.2% TFA and 0.1% FA, and ACN with 0.1–2% TFA, we found that the addition of TFA can increase the response of polymyxin, which was consistent with what has been reported [19]. When the extraction solution was pure ACN, the response and extraction recovery were low, which did not meet the quantitative requirements. Considering that TFA has ionic inhibition on MS and corrosivity, it should only be added to the extraction, and the concentration should not be high. After comparison and screening, ACN containing 0.2% TFA and 0.1% FA was selected as the extraction solution. The response and extraction recovery of PB met the analytical requirements. The extraction recovery rate was 92–99%; CV <5% (Table 1). The TFA concentration in the final analysis sample was 1.3%. TFA was only added to the sample and not to the mobile phase, which had no impact on the MS.

During the experiment, the Waters HSS T3 column (2.1 × 100 mm, 3.5 *μ*m) [20], Agilent poroshell 120 SB-C18 (2.1 × 50 mm, 2.7 *μ*m), and Waters BEH C18 (2.1 × 50 mm, 1.7 *μ*m) were evaluated. The results showed that the carry-over of Waters BEH C18 was the smallest. Therefore, this column was selected for analysis.

The entire time was 5 min, including an analysis time of 3.2 min and a posttime of 1.8 min. The retention time of PB1/PB1-I was 2.06 min; PB2/PB3 was 2.04 min. At 0–0.5 min, the aqueous phase was the main phase for keeping PB on the column and eluting substances with large polarity in the sample. In the first minute, they were almost salt. Therefore, no MS was conducted. Subsequently, the organic phase ratio was increased to elute analytes. A high organic phase (90% phase B) was used to elute impurities with low polarity. Then, returned to the initial proportion and postrun for 1.8 min to stabilize the pressure.

### 3.2. Method Validation

#### 3.2.1. Linearity and Carry-Over

Each validation or sample measurement was performed simultaneously using a standard curve. Eight points were selected to construct the standard curve, and 1/*X*^2^ was the weight factor. The correlation coefficient *R*^2^ was greater than 0.99. For PB1/PB1-I, the linearity was *y* = 1.2477*x* − 0.0214; for PB2/PB3, the linearity was *y* = 2.0285*x* + 0.0050 (Table 2). The linear range was determined by referring to the reported range of PB, the residual effect, and the actual sample concentration distribution. The concentrations of 35 samples from five patients were 100–5000 ng/mL. Therefore, the LLOQ was set at 100 ng/ml; the ULOQ was increased to 8000 ng/mL. Compared with previous methods [12, 19, 21, 22], the number of diluted clinical samples can be reduced.

#### 3.2.2. Precision and Accuracy

The intrabatch and interbatch accuracy and precision of the three batches were verified within two days. Five QC samples were selected (LLOQ, QCL, QCM1, QCM2, and QCH). The accuracy of the three intrabatch analyses was within ±15%; the CV was within 10% (Table 3). The interassay accuracy was within ±15%; the CV was within 10%, as shown in Table 3. This implies that the method is accurate and precise.

#### 3.2.3. Selectivity and Matrix Effect

The area of the analyte at the retention time in the blank sample was lower than 20% in the LLOQ. The IS area was lower than 5%, indicating high selectivity. As showed in Table 1, the matrix effect was investigated using QCH and QCL. For PB1/PB1-I, the IS‐normalized MF of QCL was 1.03 and of QCH was 1.10. For PB2/PB3, it was 1.03 and 1.05. The CV did not exceed 15% of each level, which implies that there was no matrix effect among six different batches of the normal, hemolytic, and high-fat matrix.

#### 3.2.4. Integrity of Dilution

The investigation of the two dilution factors, two and four, is presented in Table 4. The accuracy was within ±15%; the precision was within 5%. For PB1/PB1-I, the mean accuracies were 109.1 and 108.2%; the CV was 2.0 and 3.7%, respectively. For PB2/PB3, the mean accuracies were 100.6 and 101.2%; CV was 1.0 and 2.3%, respectively. Therefore, samples higher than 8000 ng/mL can be determined using dilution. The highest concentration that can be measured by this method is 32000 ng/mL.

#### 3.2.5. Stability

Stability was investigated for short-term, freeze-thaw, and long-term stability.  Table 5 shows that the sample was stable at room temperature for 4 h, at 4°C for 24 h, at −20°C, and at −80°C for three freeze-thaw cycles. The extracted supernatant was stable in an automatic sampler for 24 h. The samples were also stable when stored at −20°C for 37 days and −80°C for 97 days. The working solution was stable when stored at −20°C for 18 days. The working and stock solutions were stable at −80°C for 97 days. The stability of PB was based on the requirements of the experiments, which met the experimental requirements.

### 3.3. Clinical Application

**Figure 4:**
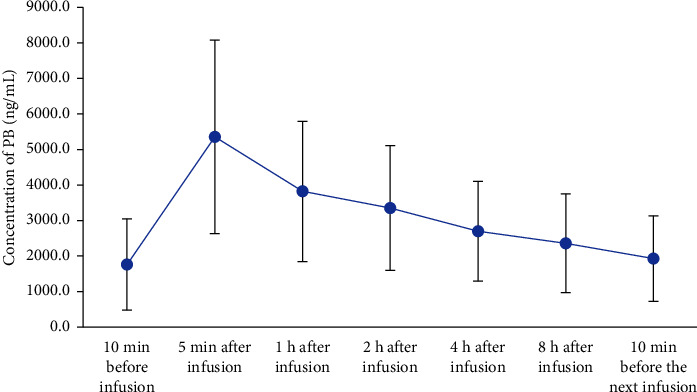
Polymyxin B metabolic trend.

The established HPLC-MS/MS method was used to measure more than 300 samples collected clinically. A steady-state metabolic curve of polymyxin B was obtained, as shown in Figure 4. This also suggests that the method can be applied to TDM and PPK.

## 4. Conclusions

In this study, a precise, accurate, and convenient method for the determination of PB was developed and validated with a linearity range of 100–8000 ng/mL for PB1/PB1-I and 11.9–948.5 ng/mL for PB2/PB3 within 5 min. To our knowledge, this is the first study to measure PB1, PB1-I, PB2, and PB3 together, which is convenient. The method was successfully applied to a PPK study of 350 samples. Therefore, it was also suitable for TDM.

## Figures and Tables

**Table 1:** Extraction recovery and matrix effect of PB.

Component	Extraction recovery			Matrix effect		
	Nominal concentration (ng/mL)	Recovery		QC level	Mean of normalized MF	CV (%)
		Mean (%)	CV (%)			
PB1/PB1-I	200	91.9	4.3	QCL	1.03	12.7
	1000	97.0	1.1			
	4000	94.3	2.7	QCH	1.10	6.4
	7000	92.1	1.9			

PB2/PB3	23.7	98.4	3.6	QCL	1.03	8.9
	118.6	99.1	0.7			
	474.3	97.6	0.9	QCH	1.05	5.3
	830.0	95.8	2.2			

**Table 2:** The calibration curve of PB.

	Batch	Slope	Intercept	*R* ^2^
PB1/1-I	1	1.3627	−0.0193	0.997
	2	1.1022	−0.0184	0.996
	3	1.2782	−0.0264	0.998

Average		1.2477	−0.0214	0.997

PB2/3	1	2.1410	0.0021	0.996
	2	1.8879	0.0072	0.995
	3	2.0566	0.0055	0.996

Average		2.0285	0.0050	0.996

**Table 3:** Intrabatch and interbatch precision and accuracy for PB in plasma.

Component	Nominal conc. (ng/mL)	Intraday (*n* = 5)			Interbatch (*n* = 3)		
		Found conc. (ng/mL) mean (SD)	Acc. (%)	CV (%)	Found conc. (ng/mL) mean (SD)	Acc. (%)	CV (%)
*PB1/PB1-I*							
LLOQ	100	100 (0.005)	99.9	5.4	94 (0.005)	94.0	5.0
QCL	200	188 (0.007)	94.2	3.5	190 (0.012)	94.8	6.3
QCM1	1000	934 (0.039)	93.4	4.1	953 (0.059)	95.3	6.2
QCM2	4000	3972 (0.082)	99.3	2.1	4128 (0.274)	103.2	6.6
QCH	7000	7036 (0.040)	100.5	0.6	7105 (0.328)	101.5	4.6

*PB2/PB3*							
LLOQ	11.9	12.7 (0.001)	106.8	6.6	12.7 (0.001)	107.0	6.4
QCL	23.7	24.0 (0.002)	101.3	6.6	24.7 (0.002)	104.3	7.2
QCM1	118.6	113.8 (0.002)	96.0	2.1	114.6 (0.005)	96.6	4.1
QCM2	474.3	452.4 (0.016)	95.4	3.4	466.0 (0.022)	98.3	4.7
QCH	830.0	801.0 (0.006)	96.5	0.8	793.7 (0.033)	95.6	4.2

^*∗*^accuracy = (found concentration)/(nominal concentration) *∗* 100%; ^*∗*^precision = (standard deviation)/(average of found concentration) *∗* 100%. ^*∗*^conc. is concentration; acc. is accuracy; SD is standard deviation.

**Table 4:** Integrity of dilution of PB.

Component	PB1				PB2			
	Nominal conc. (ng/mL)	Mean of found conc. (ng/mL)	Mean of Acc. (%)	CV (%)	Nominal conc. (ng/mL)	Mean of found conc. (ng/mL)	Mean of Acc. (%)	CV (%)
Dilution factor 2	10000	10905	109.1	2.0	1185.7	1192.4	100.6	1.0
Dilution factor 4	10000	10825	108.2	3.7	1185.7	1199.8	101.2	2.3

**Table 5:** Stability of PBs.

QC level	Accuracy of PB1/PB1-I				Accuracy of PB2/PB3			
	Low (%)	Medium 1 (%)	Medium 2 (%)	High (%)	Low (%)	Medium 1 (%)	Medium 2 (%)	High (%)
QC sample, room temp, 4 h	100.3	96.1	90.8	97.6	90.5	97.2	88.6	95.0
QC sample, 4°C, 24 h	104.8	107.8	111.2	106.9	107.6	108.4	109.0	103.4
Auto sampler, 4°C, 24 h	106.8	104.6	101.0	98.9	110.4	105.3	112.0	105.9
Freeze–thaw, −20°C, 3 times	88.8	85.7	87.4	88.6	96.8	91.0	92.3	92.3
Freeze–thaw, −80°C, 3 times	106.5	90.9	97.1	101.4	105.4	101.1	96.2	103.3
QC sample, −20°C, 37 days	113.9	104.3	105.0	113.1	103.6	96.8	99.3	104.6
QC sample, −80°C, 94 days	102.5	105.0	105.4	107.3	109.2	106.2	104.7	105.2
Working solution, −20°C, 18 days	110.2	105.0	105.6	110.6	95.8	101.3	99.7	101.5
Working solution, −80°C, 94 days	91.6	96.3	103.3	99.3	98.5	95.2	101.0	96.9
Store solution, −80°C, 94 days	92.8	98.5	97.3	95.1	92.9	95.9	96.5	96.3

## Data Availability

The data used to support the findings of this study are included within the article.
